# Providing evidence-based knowledge on nursing interventions at the point of care: findings from a mapping project

**DOI:** 10.1186/s12911-022-02053-8

**Published:** 2022-11-28

**Authors:** Renate Ranegger, Simon Haug, Janine Vetsch, Dieter Baumberger, Reto Bürgin

**Affiliations:** 1 Department of Research and Development, LEP AG, 9000 St. Gallen, Switzerland; 2 Institute of Medical Informatics, UMIT – Private University for Health Sciences, Medical Informatics and Technology, 6060 Hall in Tirol, Austria; 3 Department of Health, Institute of Applied Nursing Science, Eastern Switzerland University of Applied Sciences, 9000 St. Gallen, Switzerland

**Keywords:** Evidence-based knowledge, Nursing interventions, Point of care, Integrative knowledge management system

## Abstract

**Background:**

In healthcare there is a call to provide cost-efficient and safe care. This can be achieved through evidence-based practice (EBP), defined as the use of evidence from research, context, patient preferences, and clinical expertise. However, the contemporary and process-integrated supply of evidence-based knowledge at the point of care is a major challenge. An integrative knowledge management system supporting practicing clinical nurses in their daily work providing evidence-based knowledge at the point of care is required. The aim of this study was (1) to map standardized and structured nursing interventions classification and evidence on a knowledge platform to support evidence-based knowledge at the point of care, and (2) to explore the challenge of achieving interoperability between the source terminology of the nursing interventions classification (LEP Nursing 3) and the target format of the evidence provided on the knowledge platform (FIT-Nursing Care).

**Methods:**

In an iterative three-round mapping process, three raters, nurses with clinical and nursing informatics or EBP experience, matched nursing interventions from the LEP Nursing 3 classification and evidence provided from Cochrane Reviews summarized on FIT-Nursing Care as so-called study synopses. We used a logical mapping method. We analysed the feasibility using thematic analysis.

**Results:**

In the third and final mapping round, a total of 47.01% (252 of 536) of nursing interventions from LEP Nursing 3 were mapped to 92.31% (300 of 325) of synopses from FIT-Nursing Care. The interrater reliability of 77.52% suggests good agreement. The experience from the whole mapping process provides important findings: (1) different content orientations—because both systems pursue different purposes (content validity), (2) content granularity—differences regarding the structure and the level of detail in both systems, and (3) operationalization of knowledge.

**Conclusion:**

Mapping of research evidence to nursing classification seems feasible; however, three specific challenges were identified: different content orientation; content granularity; and operationalization of knowledge. The next step for this integrative knowledge management system will now be testing at the point of care.

## Background

Healthcare workers are expected to provide the best possible care for each patient. The growing complexity resulting from longer life expectancy and increasing number of patients, along with continuously rising healthcare costs, is creating a demand for high quality standards and efficiency in nursing practice [1]. Nursing professionals are legally obligated to make the nursing process “efficient, appropriate and cost-ffective” [2]. At the same time, patients expect the use of safe and effective interventions and avoidance of unnecessary therapies. The method of evidence-based practice (EBP) tries to meet these demands. Evidence-based practice is important to improve the quality of patient care, enhance nursing practice, and increase the confidence in decision-making. Integrating evidence into practice is expected to influence nurses’ perceptions of integrating scientific evidence into the steps of the nursing process not as a laborious chore, but as an intuitive source of knowledge. The body of evidence-based knowledge is expanding exponentially. However, the contemporary and process-integrated supply of evidence-based knowledge at the point of care is a major challenge. Scientific evidence is often given insufficient consideration. The reasons for the lack of integration of scientific findings and the application of EBP are well known; they include: insufficient EBP knowledge and skills, lack of EBP mentors and facilitators, perceptions that EBP takes too much time, and organizational cultures and environments that do not support EBP, but also insufficient time, limited access and financial resources and a lack of organizational guidelines for implementing evidence-based care [3].

To address some of the access and time issues, a knowledge management system integrated in the electronic health record enabling the support of healthcare professionals in their work by providing evidence-based knowledge at the point of care could be established. Knowledge management is a broad and multi-faceted topic involving socio-cultural, organizational, behavioural, and technical dimensions. The knowledge management framework by Alavi and Denford is based on the view of organizations as knowledge systems that include four knowledge processes: creation, storage and retrieval, transfer, and application. *Knowledge creation* refers to the development of “new” organizational know-how and capability. *Knowledge storage and retrieval* refers to the development of organizational memory (i.e., stocks of organizational knowledge) and the means for accessing its content [4]. *Knowledge transfer* takes a source-and-recipient view [5]. *Knowledge application* refers to the use of knowledge for decision-making, problem-solving, and coordination by individuals and groups in organizations [4]. Integrated knowledge management has been shown to improve the knowledge acquisition of nurses, management activities of decision makers to facilitate sustained and effective evidence-based practices, and provide easy access to evidence-based knowledge. To our knowledge no knowledge management system integrated in the electronic health record exists so far in healthcare at the point of care.

In this study, we focused on the electronic knowledge transfer between codified nursing interventions and evidence-based knowledge to provide practical knowledge (know-how) through research results [6]. The concept of interoperability is a way to provide fundamental linkage, integration, and meaningful use of healthcare data between systems, organizations, and users [7]. Based on a structured and standardized nursing interventions classification, evidence-based nursing knowledge can be offered locally and timely at the point of care through mapping. According to the “collect once, use many times” paradigm [8], standardized and structured nursing interventions in electronic documentation enable the reuse of data from the nursing process for multiple purposes such as direct access at the point of care to practical knowledge within the context of knowledge management (e.g., guidelines, quality standards).

As a nursing interventions classification, we used LEP Nursing 3 (LEP = Leistungserfassung in der Pflege, “documentation of nursing activities”; i.e., nursing workload measurement). LEP Nursing 3 is used in the electronic health record and allows statistical evaluations of nursing data [9].

For evidence-based knowledge, we used synopsis of Cochrane reviews from FIT-Nursing Care. FIT-Nursing Care is a digital platform that summarizes and critically evaluates international research knowledge in nursing and makes it available in German [10].

The lack of ability to transfer knowledge to the point of care is a key detriment for healthcare organizations’ realization of the full value of their knowledge assets. The aim of this study was:

1. to map a standardized and structured nursing interventions classification and a knowledge management system to support evidence-based knowledge at the point of care, and
2. to explore the challenge of semantic interoperability when mapping between LEP Nursing 3 and FIT-Nursing Care Cochrane Review synopses.

## Methods

### Mapping process

We chose a logical mapping method. This is a mapping method designed to match a specific FIT synopsis (target system) with the corresponding terminology of the nursing interventions classification LEP Nursing 3 (source system) based on a logical combination. The term and definition of intervention was examined to see whether it captured the meaning of the FIT synopsis (logical match).

The source system of this mapping, LEP Nursing 3, is currently being used in the electronic medical records in over 800 healthcare organizations in Germany, Austria, and Switzerland. It allows structured care planning and documentation (primary use) and statistical evaluations of nursing data (secondary use), together with nursing workload measurements [9]. LEP Nursing 3 is semantically based on the International Classification for Nursing Practice (ICNP) [11] and is structured according to ISO 18104:2014 [12]. The LEP categories for the services provided by healthcare professionals are arranged according to hierarchical criteria based on levels in a monohierarchical structure. Four service types are distinguished: (1) main service group; (2) service group; (3) service subgroup, and (4) nursing interventions. For example, multiple interventions are merged into a single service subgroup, or multiple service subgroups are merged into a single service group [9].

The target system, FIT-Nursing Care, offers nurses access to translated international scientific study synopsis from Cochrane Reviews, for example, refers to (inter)national guidelines, and performs literature searches to answer clinical questions from different institutions. The aim of FIT-Nursing Care is to promote evidence-based practice amongst nurses in a variety of settings and to provide healthcare professionals with evidence-based information as a basis for their decision-making in practice [10].

For the mapping, three raters—nurses with clinical and LEP or FIT-Nursing Care expert knowledge—were recruited. First, a training session was held to brief the raters on the mapping method. The first mapping round consisted of Rater A (RR) and Rater B (CS), the second mapping round of Rater B (CS) and Rater C (SH), and the third mapping round of Rater A (RR) and Rater C (SH). The mapping lasted from September 2018 to February 2020. The raters matched a total of 536 LEP nursing interventions to 325 FIT synopses from Cochrane Reviews (see Table 1).

**Table 1:** Raters and dates for each mapping round

	Rater 1	Rater 2	Date
Mapping Round 1	A	B	September 2018
Mapping Round 2	B	C	November 2019
Mapping Round 3	A	C	February 2020

To ensure consistency with mappings, uncertainties concerning special mapping rules were discussed and solved after mapping round 1 and mapping round 2. The resulting discrepancies, e.g. the different mapping result between nine nursing interventions *(Performing mobility training, Dispensing guidance/instruction, Dispensing advice, Dispensing advice on adherence/compliance, Dispensing information, Executing telephonic consultation, Conducting a feedback discussion, Conducting a motivational discussion, Implementing behaviour training)* to FIT synopsis *(Promoting patient uptake and adherence in cardiac rehabilitation)* were clarified in a subsequent consensus process by consulting a nurse with FIT-Nursing Care expert knowledge.

### Intra- and interrater reliability

All mapping rounds were recorded in an access database and were analysed using R statistics [13]. As the mapping of LEP to FIT includes 1:n matches, interrater reliability was assessed using a specific defined metric. The metric for each mapped concept ranged from 0 (no match) to 1 (full match). The following scores were assigned: (a) In the case of identical mapping, including the cases where the rater did not find a mapping, the agreement score was set to 1; (b) in cases of partial agreement or in the case where one rater did not find a mapping but the other rater did, a score of 0.5 points was assigned; and (c) if the ratings were completely distinct, 0 points were assigned. Intra- and interrater reliability were defined as the average of these scores.

### Identified challenges of semantic interoperability

Discussions regarding the mapping rules and the consensus process were collected according to the method of an unstructured observation in which a researcher (RR) took field notes. After structuring the field notes, they were analysed according to the method of thematic analysis [14]. A researcher (RR) systematically coded the qualitative data using a coding manual. Two researchers (RR, SH) reviewed, defined, and named themes and reported the code categories [15]. The process was documented and the results were determined by consensus by the research team (RR, SH, JV, DB).

## Results

### Mapping results, intra- and interrater reliability

A total of 52.8% (283/536), 45.5% (244/536), and 47.0% (252/536) of the LEP Nursing 3 interventions could be matched to 96.0% (312/325), 94.5% (307/325), and 92.3% (300/325) of the FIT synopses. In the consensus, 33.6% (180/536) of the LEP Nursing 3 interventions could be mapped to 78.2% (254/325) of the FIT synopses (see Table 2).

**Table 2:** Matched LEP nursing interventions and FIT synopses per mapping round

	Matched LEP Nursing 3 Interventions		Matched FIT Synopses	
Mapping Round 1	283/536	52.80%	312/325	96.00%
Mapping Round 2	244/536	45.52%	307/325	94.46%
Mapping Round 3	252/536	47.01%	300/325	92.31%
Consensus	180/536	33.58%	254/325	78.15%

The frequency of assignment of LEP Nursing 3 interventions to FIT synopses are highly variable. For example, while 72.7% (24/33) interventions in the service group *Medication* could be mapped, no nursing interventions could be mapped from the service group *Chaperoning/support.* In the LEP Nursing 3 service group *Nutrition* 1.8 FIT synopses could be mached per intervention, while in the LEP Nursing 3 service group *Education/dialogue* 8.7 FIT synopses could be mached per intervention (see Table 3).

**Table 3:** 14 LEP Nursing 3 service groups, interventions per service group, matched interventions per service group, matched FIT synopses, and average matched FIT synopses per LEP Nursing 3 service group

LEP Nursing 3—service groups	Interventions per service group	Matched interventions per service group		Matched FIT synopses	Average matched FIT synopses
Movement	49	22	44.90%	106	4.8
Personal care/dressing	43	10	23.26%	14	1.4
Nutrition	17	12	70.59%	22	1.8
Excretion	30	7	23.33%	25	3.6
Respiration/circulatory system	18	4	22.22%	7	1.8
Education/dialogue	32	21	65.63%	183	8.7
Activity	40	15	37.50%	114	7.6
Chaperoning/support	12	0	0.00%	0	0
Safety	85	24	28.24%	43	1.8
Laboratory testing	34	4	11.76%	5	1.3
Medication	33	24	72.73%	105	4.4
Treatment	105	29	27.62%	102	3.5
Review	12	6	50.00%	16	2.7
Documentation/organisation	26	2	7.69%	3	1.5
Total	536	180		745	

Table 4 gives an overview of the distribution of the number of FIT synopses per LEP nursing intervention. Around 100 LEP nursing interventions could be matched exactly to one FIT synopsis (1:1 match). For example, the nursing intervention *“Performing bladder irrigation”* corresponds exactly to the FIT synopsis *“Irrigation methods of long urinary catheters in adults”* [16]. About 150 LEP nursing interventions have a 1:n relation ranging from 1:2 to 1: > 20. In the consensus, six LEP nursing interventions were assigned to more than 20 FIT synopses.

**Table 4:** Distribution of the number of FIT synopses per LEP nursing intervention

	Total LEP Nursing Interventions	No match	1:1 match	1:n match	2–5	6–10	11–15	16–20	> 20
Mapping Round 1	536	253	96	187	110	28	21	12	16
Mapping Round 2	536	292	95	149	88	24	13	10	14
Mapping Round 3	536	284	99	153	96	27	14	5	11
Consensus	536	356	72	108	74	22	4	2	6

**Fig. 1:**
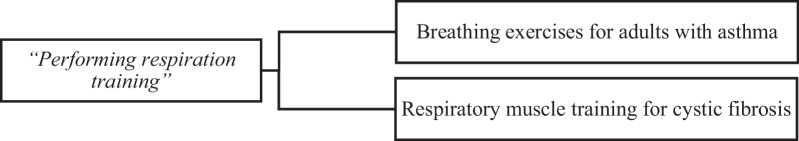
Extract from the mapping between the LEP nursing intervention *“Performing respiratory training”* and two FIT synopses

Figure 1 shows as an example of an extract from the mapping between the LEP nursing intervention *“Performing respiratory training”* as source format to two FIT synopses *“Breathing exercises for adults with asthma”* [17] and *“Respiratory muscle training for cystic fibrosis”* [18] as target format.

The agreement between the raters per round, considering all LEP nursing interventions (n = 536), was 71.7%. Table 5 shows the interrater reliability. At 77.4%, the agreement was particularly high in mapping round 3 between Rater A and Rater C. If LEP interventions without assignment were excluded, the agreement dropped to around 40%. Agreement was particularly high (50.4%) in round 3 and particularly low (28.6%) in round 1.

**Table 5:** Interrater reliability by 3 raters and 3 mapping rounds (columns 1–3: including 536 LEP nursing interventions; columns 4–6 excluding LEP nursing intervention without assignment)

Interrater reliability (including all 536 LEP nursing interventions)			Interrater reliability (excluding LEP nursing intervention without assignment)		
Round 1	Round 2	Round 3	Round 1	Round 2	Round 3
(334/536) 62.31%	(405/536) 75.55%	(415/536) 77.42%	(81/283) 28.62%	(102/233) 43.77%	(123/244) 50.40%

### Identified challenges

During the mapping, we identified potential challenges to semantic interoperability within the content perspective:

#### Different content orientations

The mapping provided important findings for further content development for both systems, i.e., LEP nursing interventions and FIT-Nursing Care synopses. For example, phytotherapy, telenursing, and family nursing are tasks that may become more important in nursing in the future. Missing LEP nursing interventions related to these topics indicate a need for evaluation as part of the systematic LEP release management and, depending on the result, interventions are integrated into the new version according to the classification principle. Topics such as pregnancy or quitting smoking are relatively overrepresented in FIT-Nursing Care. Topics such as oncological care, palliative care, delirium/dementia, and psychiatric care were underrepresented or not represented at all. The mapping process also raised the question of which nursing interventions require evidence-based explanations. For example, the nursing intervention *“Performing a full body wash”* could not be assigned to any FIT synopsis.

#### Content granularity

During the mapping process, we observed differences regarding the structure and the level of detail in both systems. While some LEP interventions have a high degree of aggregation, such as “*Preparing/adapting auxiliary aids*”, the linked FIT synopsis *“Hip protectors for preventing hip fractures in older people”* is very detailed. Conversely, other LEP interventions have a high level of detail. For example, the nursing intervention “*Providing advice on diabetes management*” is very detailed in the sense of a nursing intervention. In contrast, the linked FIT synopsis *“Education programmes for people with diabetic kidney disease”* is aggregated regarding the intervention (education programme), but very specific in connection with the status picture (people with diabetic kidney disease). Therefore, it was sometimes unclear whether the described intervention of the FIT synopses corresponded exactly to the LEP intervention and vice versa making it difficult at times to clearly match LEP interventions to FIT synopses.

#### Operationalization of knowledge

Certain topics, such as pregnancy or smoking cessation, are overrepresented, while other topics were underrepresented (e.g., oncological care, palliative care, delirium/dementia, psychiatric care). Furthermore, it was found that some synopses focus on a medium (e.g., telephone), concepts (e.g., Case management approaches to home support for people with dementia) or organization-specific factors (e.g., Admission avoidance hospital at home) rather than the actual nursing interventions. Telephonic consultation, telemonitoring or case management potentially could be interpreted as a nursing intervention that is part of clinical care post-discharge. Concepts describe nursing interventions according to ISO 18104:2014. Consequently, these synopses often could not be mapped to a nursing intervention.

## Discussion

The aim was to map nursing interventions classification (LEP Nursing 3) and synopses on a knowledge platform (FIT-Nursing Care) to support evidence-based knowledge at the point of care and to explore the challenge of achieving interoperability between those two systems. We were able to map a total of 47.01% of nursing interventions from LEP Nursing 3 to 92.31% of synopses from FIT-Nursing. Most of the interventions could be mapped to FIT synopses in the LEP Nursing 3 service group *Medication*, *Nutrition*, and *Education/dialogue*. In the service group *Education/dialogue*, there could be mached 8.7 FIT synopses per intervention. This high proportion could be related to the fact, that empowerment issues have recently gained a lot of attention in many countries.

Our study provided an initial basis for the implementation of an integrative knowledge management system based on LEP Nursing 3 and FIT-Nursing Care; however, some challenges need to be addressed. Challenges which were encountered during the mapping process included: the different **content orientations**, differences in terms of **structure** and level of detail in both systems, and **operationalization of knowledge**.

Given the need to provide evidence-based knowledge at the point of care, establishing an integrative knowledge management system is important for the further development of healthcare professionals. The mapping based on LEP Nursing and FIT-Nursing Care built a solid base to test the feasibility of an integrative knowledge management system to provide evidence-based knowledge at the point of care. We know from studies [e.g. 19] that gaps continue to exist between what is known and what is done in practice. Nurses often cited the lack of time and knowledge as main factors that keep them from using evidence-based practice at the point of care. Having a knowledge system at the point of care could reduce these barriers. The proposed mapping would allow future software applications to obtain easy access to evidence-based knowledge (know-how) in the care plan based on a nursing intervention. An integrative knowledge management system should focus not only on the technique’s know-how but also on “know-why” and “know-what” [20]. The mapping currently does not consider these two components of knowledge (know-why, know-what), but we are considering developing such a project after a first feasibility study in a clinical setting.

Two thirds of the LEP Nursing 3 interventions could not be mapped to FIT synopses. These included nursing interventions which were not contained in FIT synopses. It is questionable if this is an actual research gap or whether there is no need to test such interventions in research studies. Due to the different development-related **target orientations** of the two systems, we reported a different **content granularity**. Therefore, a clear assignment of the nursing interventions to the FIT synopses was not always possible and some LEP interventions were linked to over 20 FIT synopses.

In addition, the over- and underrepresentation of certain topics, as well as synopses that focus on the medium instead of the intervention, further complicated the mapping process. Regarding the further development of the content of FIT-Nursing Care, the utility and use of Cochrane Reviews which focus on the media or organization specific factors rather than the actual nursing interventions should be discussed. Such synopses seem particularly useful for professionals involved in the development of healthcare services. However, with regard to everyday nursing care, the focus of FIT synopses should be on nursing interventions in the future.

Cochrane Reviews were chosen for this mapping process because they are currently considered the gold standard and because they also receive updates from time to time. This aspect is central to ensuring that the evidence is up to date. Nevertheless, the mapping process showed that the high level of detail of the Cochrane Reviews sometimes makes it difficult to clearly assign evidence to nursing interventions and that the restriction to Cochrane Reviews reduces the pool of evidence accordingly.

An ongoing expansion of the evidence in the system can be undertaken, for example, by targeted screening for relevant studies; for this purpose, inclusion or exclusion criteria must be established and the potential gaps in the data base must be known. Semi(automatically) supported screening can also be helpful here. Future work is still needed to determine what steps can be taken to expand the data base, include studies with specific interventions, and also keep the evidence up to date.

Before testing the mapping in a feasibility study, the mapping should be reviewed by practitioners to identify evidence which is relevant for practice and reduce the linkages. We also could do a needs assessment with nurses to ask, what they would actually need from an informatics and decision-making perspective at the point of care. A further challenge was the time needed to map the synopses manually to the LEP interventions. Before implementation into practice, it would need to be established how the evidence body can be expanded and kept up to date without manually spending hours on updating the evidence for each intervention. Automated terminology mapping approaches could be used in future studies to increase the list of possible matches. Multiple mapping methods such as automated terminology mapping approaches could support the rate of equivalence [21].

### Strengths and limitations

Overall, our mapping created a theoretical basis to establish an integrative knowledge management system, even if the study has some limitations: First, the mapping was carried out theoretically by experts and there are many 1:n mappings. Therefore, we do not know whether the available mapping results cover the actual knowledge need of nursing practice. Second, the interrater and intrarater reliability in this study is moderate. While interrater and intrarater reliability is an important element in reliability testing of an instrument, it should be noted that this is only one of several indicators. Another indicator, for example, is the validity testing [22]. However, in our context, the reliability test evaluated the objectivity and the stability of the mapping. This means that the mapping decisions were entirely dependent on the researchers. However, a test phase will answer questions regarding the objectivity, reliability, practicality, and applicability of this mapping. A further limitation was that only a subselection of FIT synopses (n = 325) was mapped to the LEP interventions. Therefore, we cannot draw a conclusion on missing evidence as such. However, it provides a basis for the selection of reviews where evidence was lacking so far.

## Conclusions

The results of this study have highlighted a way in which evidence-based knowledge could be made accessible mapped to nursing interventions at the point of care. This is an important finding to create the basis for a knowledge management system integrated in an electronic health record. As a next step, this proposed mapping needs to be implemented in a software application. Thus, it could be tested in a clinical setting before being introduced in nursing practice. The testing will also show whether the mapping can adequately support nurses in daily nursing interventions or if additional information elements such as nursing assessments, nursing diagnoses, or medical diagnoses need to be considered in future mappings. The key question is the feasibility of this integrative knowledge management system.

### Implications for nursing practice

This knowledge management system, integrated within an electronic health record, could support clinical decision making and optimize patient outcomes. It may help nurses select and implement appropriate, evidence-based interventions at the point of care.

## Data Availability

The datasets generated and/or analysed during the current study are not publicly available due license terms but are available from the corresponding author on reasonable request.
